# Antimicrobial Impact of Wood Vinegar Produced Through Co-Pyrolysis of Eucalyptus Wood and Aromatic Herbs

**DOI:** 10.3390/antibiotics13111056

**Published:** 2024-11-06

**Authors:** Gil Sander Próspero Gama, Alexandre Santos Pimenta, Francisco Marlon Carneiro Feijó, Caio Augusto Martins Aires, Rafael Rodolfo de Melo, Caio Sérgio dos Santos, Lúcio César Dantas de Medeiros, Thays Vieira da Costa Monteiro, Maíra Fasciotti, Priscila Lira de Medeiros, Maria Rita Macêdo de Morais, Tatiane Kelly Barbosa de Azevedo

**Affiliations:** 1 Graduate Program in Forest Sciences—PPGCFL, Universidade Federal do Rio Grande do Norte, Rodovia RN 160, km 03 s/n, Distrito de Jundiaí, Macaíba 59280-000, RN, Brazil; gil.gama@ifpi.edu.br (G.S.P.G.); lucio.cesar@ufrn.br (L.C.D.d.M.); priscila.lira.089@ufrn.edu.br (P.L.d.M.); maria.morais.708@ufrn.edu.br (M.R.M.d.M.); tatiane.azevedo@ufrn.br (T.K.B.d.A.); 2 Graduate Program in Environment, Technology, and Society—PPGATS, Universidade Federal Rural do Semiárido—UFERSA, Av. Francisco Mota, 572—Bairro Costa e Silva, Mossoró 59625-900, RN, Brazil; marlon@ufersa.edu.br (F.M.C.F.); rafael.melo@ufersa.edu.br (R.R.d.M.); caiosergio@ufersa.edu.br (C.S.d.S.); 3 Departament of Health Sciences, Universidade Federal Rural do Semiárido—UFERSA, Av. Francisco Mota, 572—Bairro Costa e Silva, Mossoró 59625-900, RN, Brazil; caio.aires@ufersa.edu.br; 4 Laboratory of Organic Analyses, Instituto Nacional de Metrologia, Qualidade e Tecnologia, Av. Nossa Sra. das Graças, 50, Xerém, Duque de Caxias 25250-020, RJ, Brazil; tvmonteiro@inmetro.gov.br (T.V.d.C.M.); mfasciotti@inmetro.gov.br (M.F.)

**Keywords:** adjuvant compounds, microbial resistance, natural antimicrobials, wood carbonization liquids

## Abstract

Background: The search for substances that can overcome microorganisms’ resistance and enhance the antimicrobial activity of given products has attracted the attention of researchers. Eucalyptus wood vinegar (WV) is a promising product for developing alternative antimicrobials. Objectives: This study aimed to evaluate whether the production of WV in the co-pyrolysis of eucalyptus wood with aromatic herbs would incorporate compounds from them into WV and if that would enhance its antimicrobial action. Methodology: WV was produced alone and through co-pyrolysis with marjoram (*Origanum majorana*), Peruvian oregano (*Origanum vulgare*), rosemary (*Salvia rosmarinus*), thyme (*Thymus vulgaris*), and Turkish oregano (*Origanum onites*) at a proportion of 25% of herbs to the bone-dry wood weight. The antimicrobial effects were assessed against strains of gram-negative and -positive bacteria, and *Candida glabrata*. Microorganisms’ colony growth in agar had their absorbances recorded after inoculation and incubation. Chemical characterization of the new products was performed by gas chromatography and mass spectrometry (GC/MS). Results: After coproduction, there were relevant chemical changes concerning the original WV. Thymol, for instance, was incorporated into the WV through co-pyrolysis with marjoram, Peruvian and Turkish oregano, and thyme. The coproducts were more efficient than the WV produced only with wood, with thyme-incorporated products having the highest efficiency. This can be attributed to the increase and incorporation of the substances after coproduction, and particularly the role of thymol in enhancing the antimicrobial action. Conclusion: Given the results, the co-production of WV with eucalyptus wood and aromatic herbs has the potential to provide alternative antimicrobial products.

## 1. Introduction

The World Health Organization has recognized that microbial drug resistance has become one of the most significant global public health crises [1]. The emergence of this resistance is attributed to the excessive and inappropriate use of antibiotics, both in human medicine and in animal production, playing a central role in the increased emergence and spread of resistant microbial strains [2,3,4]. Microbial resistance is microorganisms’ ability to survive and multiply in the presence of drugs previously capable of inhibiting them [1,5]. This resistance can severely reduce the efficiency of conventional treatments, making it difficult to cure diseases, increasing mortality rates, and, in addition, generating several negative impacts on clinical procedures such as surgeries and wound healing which depend on the use of antimicrobial drugs to prevent infections [6,7]. Furthermore, microbial resistance increases hospital costs [8]. The World Health Organization and World Bank estimate additional healthcare costs worth US $1 billion by 2050 due to the resistance of microorganisms to antimicrobials [1].

Under such circumstances, there is an imminent need to search for efficient therapeutic alternatives as an urgent priority. Thus, among the other options researched, plant-based compounds have proven to be promising products [9]. This is mainly due to various chemical components with diverse bioactive effects present in these products, including phenolic compounds, aldehydes, phenols, and organic acids [10,11]. Unlike conventional antibiotic drugs, which generally have only a single target in microorganisms, plant-based products often act on multiple target sites in cells, reducing the chance of resistance emerging [12,13]. Thus, the promising potential that natural products have in the face of the reality of the emergence of microbial resistance and the global health crisis that this situation determines becomes evident. A product of natural origin with promising bioactive potential is eucalyptus WV, derived from the pyrolysis processes. This production occurs primarily through wood carbonization or slow pyrolysis, the raw material’s thermal degradation process in the controlled presence of oxygen [14]. During charcoal production, part of the vapors released from the material through smoke (approximately 33 to 57%) can be condensed, producing the raw WV, from which refined WV can be made by vacuum or single distillation [15,16,17,18].

Wood vinegar, also known as pyroligneous acid, is the product corresponding to the aqueous fraction of pyrolysis liquids. It has an acidic character and a rich and varied chemical composition [19,20,21]. Several studies report the wide range of compounds present in the chemical constitution of EP, with studies demonstrating the occurrence of more than 100 compounds, emphasizing the considerable presence of chemical groups such as phenolic compounds, aldehydes, phenols, organic acids, etc. [14,22,23,24,25,26,27]. This chemical particularity has brought special attention to WV for application in various research worldwide. Several studies explore WV’s bioactive potential, demonstrating promising results. Among the investigations carried out regarding the bioactivity of WV are those investigating its anti-inflammatory action [28], antiviral [29], antioxidant [30], and healing properties [31], its role as an animal growth promoter [32], and its plant growth [33], insecticide [34], herbicide [14], and antimicrobial properties [4,21].

Having demonstrated the versatility of WV as a bioactive product, the present research focuses on its antimicrobial potential. Its application as a potent alternative to inhibit microbial growth is already established in the literature [35]. The present research results from a series of studies investigating the use of WV in controlling bacteria and fungi growth. Previous studies consolidate eucalyptus EV as an effective antimicrobial [27,36,37,38,39]. However, these studies evaluated WV produced only with eucalyptus wood. Recently, the possibility of enhancing the inhibitory effect of WV with essential oils from herbs came to light. To verify if such a strategy is promising, this study tests new types of WV produced through the co-pyrolysis of wood and herbs that effectively aggregate bioactive chemical substances capable of acting as antimicrobials or adjuvants. In the present context, an adjuvant is a substance that can increase the effectiveness of other antimicrobial products through synergistic action between them [40]. Adjuvants have been investigated as a promising strategy to combat the problem surrounding the emergence of microbial resistance, especially against multidrug-resistant strains [41]. Plant extracts and essential oils have aroused great interest as alternatives to conventional antimicrobials, mainly due to their rich chemical composition, which includes phenolic compounds, terpenes, and flavonoids. These substances can have their own action or function as essential adjuvants [42,43,44,45].

Among the most studied vegetables are *Origanum majorana* (marjoram), *Origanum vulgare* (Peruvian oregano), *Origanum onites* (Turkish oregano), *Rosmarinus officinalis* (rosemary), and *Thymus vulgaris* (thyme). These vegetables are known for their essential oils, rich in several bioactive compounds, such as thymol, carvacrol, eucalyptol, and others, which can interact with the cell membrane of microorganisms, cause oxidative stress, and inhibit microbial growth [46,47,48,49,50,51,52]. Using these extracts and essential oils offers a promising approach to controlling bacterial infections without the side effects associated with the overuse of synthetic antibiotics. However, the large-scale application of these natural extracts faces considerable challenges, mainly due to the high extraction cost and the large amount of plant material required to produce small volumes of extracts or essential oils [53,54,55,56,57,58,59]. Extraction by steam distillation or pressing requires large amounts of biomass, which may limit their economic viability for large-scale commercial use [57,58]. These barriers challenge advancing the industrial application of essential oils as natural antimicrobials, requiring innovations in extraction technologies to reduce costs and improve production efficiency.

Recent research works focused on thymol, found in essential oils of *Thymus vulgaris, Origanum vulgare*, and *Origanum majorana*, for example, popularly known as thyme, Peruvian oregano, and marjoram [53,55,56,60,61,62]. Some studies consolidate thymol as an agent that enhances the action of antimicrobial drugs [45,63]. It is believed that this substance has this potential because it acts on the cell membrane of microorganisms [64], which could result in an excellent option for combined use with other products, aiming to enhance them. Eucalyptus wood vinegar does not contain thymol or any other adjuvant cited above in its composition [24,27]. Therefore, it is worth investigating incorporating these compounds into WV via the coproduction of WV combining wood and aromatic plants.

Thus, this study aimed to evaluate whether the co-pyrolysis of eucalyptus wood and aromatic herbs can incorporate substances to WV, such as thymol and others, into its chemical composition and enhance the antimicrobial action of this product. In addition, through a thorough review of the literature, this manuscript aimed to shed some light on the potential of co-pyrolysis of eucalyptus wood with herbs as a source of natural antimicrobials, with our experimental results as a basis for comparison.

## 2. Results

### 2.1. Carbonization and Pyrolysis Liquids Refining

**Figure 1:**
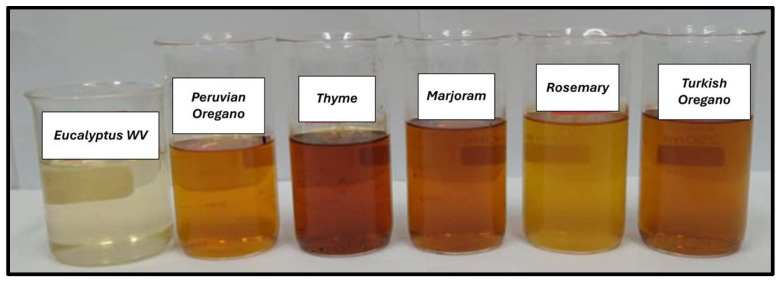
Types of WV produced and assessed in the experiment.

Figure 1 displays the types of WV produced and assessed in this experiment and highlights the differences between the color of the products. All of the products are crystalline after refining. While WV from eucalyptus wood presented a light yellow color, the others tended to darker shades, showing the influence of the essential oils from the aromatic herbs.

Table 1 displays the carbonization run results for eucalyptus wood and its combinations with aromatic herbs. The results obtained for WV from the co-pyrolysis of wood plus aromatic herbs are close to the gravimetric yields observed for the carbonization of eucalyptus wood only. When herbs were combined with wood, the yield in the pyrolysis liquids did not increase significantly, with the same pattern for the gases, when compared to the witness treatment (T1—wood only). This pattern is consonant with results cited in the literature for eucalyptus clones [16,24,65] and also in industrial carbonization [27,66]. In this experiment, all treatments presented yields in raw pyrolysis liquids higher than 40%. From an industrial standpoint, this is interesting, because when charcoal and liquid yields are maximized, the economic balance is favorable since they are saleable products. Still, the yields in purified WV obtained from the raw pyrolysis liquids by vacuum refining presented no difference for the liquids produced from wood alone and wood co-pyrolyzed with herbs. For all of them, the refining results were roughly higher than 95%, which is consonant with the results previously reported [37].

### 2.2. Chemical Profiling of WVs

From the chemical profiling standpoint, the products’ composition seems similar. However, when the data in Table 2 are closely analyzed and compared, significant differences exist between the WVs produced with wood only and those made with wood and herbs. Compared to the WV obtained with eucalyptus wood only (T1), several compounds were reduced, emerged, and disappeared in the chemical composition of the other co-products. Table 2 lists the annotated compounds in the WVs. The combination of eucalyptus wood with aromatic herbs brought about relevant changes in the chemical composition of WV compared to the T1 treatment. Among all identified compounds, only 39 are present simultaneously in the six types of WV. For instance, the WV from the T1 treatment had 92 compounds identified, while for marjoram, Peruvian oregano, Turkish oregano, rosemary, and thyme, there were 155, 152, 139, 171, and 152, respectively. Due to the complexity of the chemical composition of the WVs, only compounds with an area percent higher than 0.03% are listed in Table 2, with a total of 129 compounds.

In addition to the general chemical composition of the products, Table 2 also presents information on the major compounds of each WV. This information can be seen in the bold markings. Major compounds were established as those that presented areas equal to or greater than 1.5%. The table shows that compounds numbered 1 to 20, 23, 27, 34, 102, and 121 were major compounds for certain WVs. Of these compounds, only four were common to the six WVs: furfural, 2-methoxy-phenol, 2-furan carboxaldehyde, 5-methyl-, and 2-cyclopenten-1-one. The WV produced only with eucalyptus wood presented 13 major compounds, of which 2-methoxy-phenol, 2,6-dimethoxy-phenol, phenol, and furfural stand out (Table 2). The product from the association of wood with rosemary presented 16 major compounds. In the products with marjoram, Peruvian and Turkish oregano, and thyme, 16, 14, 12, and 14 principal components were found, respectively.

A proper inference can be made from Table 2 regarding the most significant compounds in the six WVs’ organic fractions. The Table shows 25 major compounds with an area higher than 1.50% found in the WVs. Only four are present in all WVs: furfural, 2-methoxy-phenol, 5-methyl-2-furan carboxaldehyde, and cyclopentene. WV produced with eucalyptus wood only had 13 main compounds, standing out the 2-methoxy-phenol, phenol, and furfural. In comparison, the WV produced with rosemary had 16 significant components; the products with marjoram, Peruvian oregano, Turkish oregano, and thyme presented 16, 14, 12, and 14. Among them, the main annotated compounds were furfural (with areas from 8.92 to 23.12%), 2-methoxy-phenol (10.83 to 12.35%), 2,6-dimethoxy-phenol (0.20 to 10.30%), phenol (5.22 to 9.34%), cyclopentanone, and acetic, butanoic, propanoic acid, and others (Table 2). It is essential to highlight that some compounds that are in the majority for some WVs are in the minority for others, and this can be seen in Table 2.

Table 3 displays the values determined for MIC, MBC, and MFC for the several types of WV assessed in the present work. From the Table, it can be observed that, for some cases, the co-pyrolysis of wood and aromatic herbs positively enhanced WV’s antimicrobial activity compared to the experimental treatment in which the product was generated only from wood. For most WVs, the MIC was found to be 3.125%. However, there were lower values for some microorganisms. The most susceptible strains in the co-productions were *A. baumanni* and *P. aeruginosa*, with a MIC of 1.56% for all WVs spiked with compounds from the aromatic herbs, except with Peruvian oregano, for which all strains required MICs of 3.125%. The MIC, MBC, and MFC values were constant for the positive control test using chlorhexidine, with a concentration of 0.001%.

Comparing the other products with the WV produced only with eucalyptus wood (Table 3), it was found that the WVs enriched with compounds from aromatic herbs demonstrated greater inhibitory efficiency against most of the microorganisms tested, such as *A. baumannii*, *E. coli*, *P. aeruginosa*, *P. aeruginosa* (PA01), and *S. aureus*. In the strains where the efficiency of the co-productions did not surpass that of the WV of the wood, their inhibitory actions remained equivalent.

Comparing the WVs with the eucalyptus one (Table 3), it was found that, in general, the WVs spiked with essential oils from aromatic herbs demonstrated greater inhibitory efficiency against strains such as *A. baumannii*, *E. coli*, *P. aeruginosa*, *P. aeruginosa* (PA01), and *S. aureus* and that this efficiency remained similar against *K. pneumoniae*, *S. aureus* (MRSA), and *C. glabrata*. The MIC results obtained with the WV from eucalyptus wood did not demonstrate any inhibition at concentrations lower than 3.125%, with inhibition values between 3.125 and 6.25% WV for all strains tested (Table 3). Therefore, analyzing the data to determine which herbs were most effective, it was observed that thyme deserves to be highlighted. The co-production of WV with thyme in co-pyrolysis with eucalyptus wood revealed the lowest MIC values (1.56%) for most of the different microbial strains, namely *A. baumannii*, *P. aeruginosa*, and *P. aeruginosa* (PA01), followed by rosemary, marjoram, and Turkish oregano, which presented MIC values of 1.56%, comparable to those found for *A. baumannii* and *P. aeruginosa* (Table 3).

In contrast, the WV produced with Peruvian oregano demonstrated no effectiveness in enhancing the antimicrobial activity compared to the witness treatment. This WV did not present any MIC value below 3.125% WV, as the others did. However, this does not mean that this product was inefficient. Otherwise, it also demonstrated promising results against the strains assessed (Table 3), despite not being capable of bringing any effect superior to the WV produced with eucalyptus wood only.

**Figure 2:**
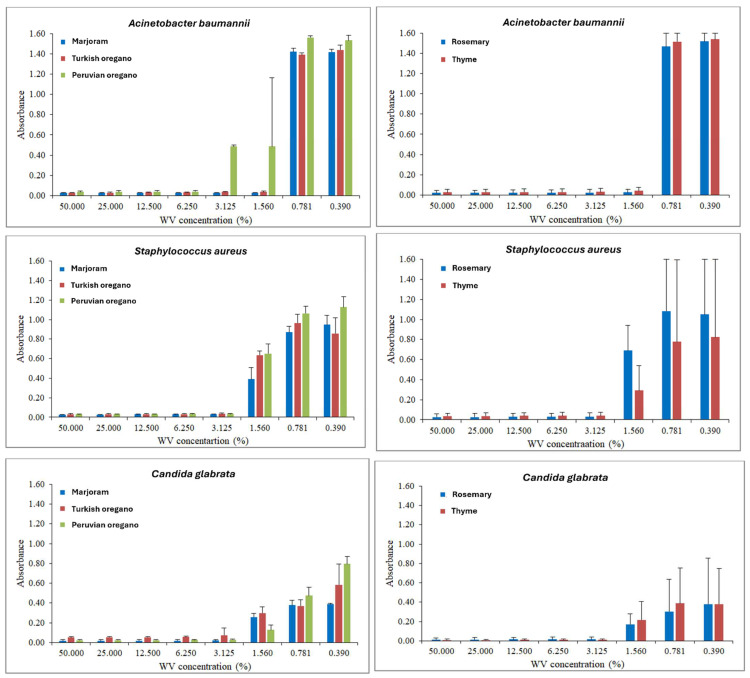
Graphs showing the behavior of absorbances *Acinetobacter baumannii*, *Staphylococcus aureus*, and *Candida glabrata* after 24 h of exposure to the different types of WV.

The absorbances demonstrating the behavior of microorganisms under the effect of exposure to different concentrations of co-produced WVs are expressed in the form of graphs (Figure 2). Three species of herbs of the same botanical genus *(Origanum onites*, *Origanum vulgare*, and *Origanum majorana*) were assessed and grouped into single graphs in Figure 2. Furthermore, due to the large number of analyses, three microorganisms were selected to present the trends found for absorbances. The selection criterion was to demonstrate the behavior of a gram-negative strain, a gram-positive strain, and a fungal strain.

All microorganisms’ absorbance values ranged from 0.01 to 0.04 right after incubation. When assessing the absorbances at the time of inoculation, all values obtained varied between 0.01 and 0.04. These low values were expected since the microbial cells had not yet multiplied. The new reading, after incubation, demonstrated an increase in these numbers. The graphs show that the highest absorbances were recorded at the lowest concentrations of extracts, which means that higher concentrations of WV resulted in lower absorbance values, demonstrating a more significant inhibitory effect of the products. The graphs indicate that after incubation, greater concentrations of WVs resulted in lower absorbance values, which shows the products’ superior inhibition capacity (Figure 2). The graphs corroborate the MIC, MBC, and MFC values that were presented before.

## 3. Discussion

### 3.1. Chemical Profiling by GC/MS

The same groups of compounds (phenolic compounds, organic acids, aldehydes, furans, etc.) remained in the resulting products through co-pyrolysis with herbs. However, significant modifications to some single compounds resulted in diversified chemical compositions (Table 2). As already highlighted, of the 306 annotated compounds, only 39 remained the same in all WVs, such as furfural, 2-methoxy-phenol, 2,6-dimethoxy-phenol, 3,5-dimethyl-phenol, butyrolactone, and pentanoic and propanoic acid, among others. Similar results regarding the general chemical composition were obtained by [14], with the predominance of 2-methoxy-phenol, phenol, 2,6-dimethoxy-phenol, and furfural. Moreover, similar to the results of [22], who determined a predominance of phenol, 2,6-dimethoxy, and 2-methoxy-phenol in WV, [25] reported that the main compound in the WV they assessed was 2-methoxy-phenol, representing 11.9% of the product they assessed. Further, [26] demonstrated a notable predominance of phenol, 2,6-dimethoxy, and 2-methoxy-phenol in the six types of WV they profiled. These comparisons indicate that the core of the chemical composition of the WVs remained stable since it depends on the kind of wood carbonized. In this work, the core of the primary compounds cited above remained even after coproduction with aromatic herbs. In the results of another study [23], the most expressive components were fatty acids: myristic, palmitic, and oleic. Those compounds predominated because they come from the carbonized material of longan fruits.

There were reductions, increases, disappearances, and emergences of specific compounds belonging to the large groups of stable compounds (phenolics, organic acids, aldehydes, etc.) of the WV (Table 2). This is evident when observing the number of components identified in each product, with 92 in the eucalyptus WV and higher values found in the WVs produced with co-productions with marjoram, Peruvian oregano, Turkish oregano, rosemary, and thyme. According to the literature, this fact was already expected since the final composition of WV depends mainly on the material used for its production [67,68,69,70,71]. This is directly linked to the macromolecular composition of this material, that is, the percentage of cellulose, hemicellulose, and lignin present in the carbonized biomass [70,72].

In the present study, adding aromatic herbs to wood in carbonization significantly altered the final composition of the WVs. These herbs have several chemical groups in their extracts and essential oils [42,53,60,73,74,75,76]. Co-pyrolyzing these materials with wood brought those chemical groups to integrate into the WV during the thermal process, modifying its final chemical composition. Thus, the essential oils were volatilized during carbonization with essential oils. Usually, thermal treatments are employed to obtain essential oils from the aromatic herbs evaluated in this work [57,77], and this may explain the transfer of chemical compounds present in herbs to the WVs, since pyrolysis is the thermal degradation of biomass in the absence of oxygen. The elimination, formation, reduction, and increase of constituents occurred after the exposure, which is expected in cases like this, as highlighted by [78,79,80,81,82,83]. This fact is evidenced, for example, regarding thymol, a chemical compound of interest which is not part of the original chemical composition of WV produced only with eucalyptus wood. However, it was observed that thymol was found in the WV after co-productions with marjoram, Peruvian and Turkish oregano, and thyme in percentages of 0.20, 0.30, 0.10, and 0.70%, respectively, with the highest value for the product from thyme (Table 2).

Another chemical compound of interest due to its antimicrobial properties was eucalyptol, present in the WVs produced through co-pyrolysis with rosemary (1.63%), marjoram (0.21%), Peruvian oregano (0.19%), and Turkish oregano (0.30%). Co-pyrolysis also extracted and incorporated several other substances into the WVs, as shown in Table 2. When checking these data, it was found that adding aromatic herbs into the carbonization seems to be a promising technological innovation for adding chemical compounds with antimicrobial activity to WV. It is an acceptable alternative since conventional methods of essential oil extraction are usually expensive and require large amounts of plant material for a relatively low yield of these oils, as pointed out by [53,54,55,56,57,58,59]. These characteristics may hinder the large-scale application of essential oils for antimicrobial treatments and significantly increase production costs. However, as found in this experiment, by adding herbs directly to the carbonization process, the bioactive substances present in the plants, such as thymol and eucalyptol, for example, can be efficiently extracted and incorporated into the WV (Table 2). Besides revealing itself as a viable alternative from a technical standpoint, the co-pyrolysis of wood with herbs can obtain sound results by using a small volume of herbs and most likely can reduce the costs associated with extraction since the WV already has in its composition a set of compounds with proven antimicrobial properties. In addition, co-pyrolysis can add value to the charcoal production chain, enhancing the use of WV for antimicrobial applications.

The co-production of WV with aromatic herbs has already been tested in the food industry to add substances that improve the smell and flavor of food products [84]. This test was carried out because WV has already been established as a preservative, flavoring, and aromatizing agent [85,86,87,88,89]. However, the co-production of WV is still unexplored in antimicrobials for veterinary and medical uses. Thus, the present research appears to be pioneering in testing some products from co-pyrolysis against pathogenic microorganisms. In this context, this discussion topic was developed in greater depth around the changes in the chemical composition of the products after the co-production process. In general, the therapeutic effect of WV has already been consolidated over the last few years [90]. Its action efficiency is attributed to its chemical constitution, rich in several bioactive compounds [24,35]. The permanence of groups of compounds, such as organic acids, phenolic compounds, furans, etc., may explain the efficiency of antimicrobial action that all tested WVs demonstrated. These large groups already have their bioactive action consolidated in the literature [91,92,93,94,95], which reinforces the antimicrobial action performed by WVs.

Despite significant differences concerning chemical composition, WVs from several lignocellulosic raw materials proved to be efficient antimicrobials against microorganisms, bacteria, and fungi [35,36,96]. However, as demonstrated herein, there were differences in the efficiency of co-products compared to the WV from wood alone. Additionally, there were differences among the co-products regarding chemical composition (Table 2). Overall, the co-products were more efficient than the WV produced only with wood against all microbial strains tested, except for *K. pneumoniae*, *S. aureus* MRSA, and *C. glabrata*. Some chemical compounds of relevant antimicrobial interest were increased in the co-produced products. Examples include furfural, cyclopentanone, 2-cyclopenten-1-one, and acetic, propanoic, butanoic, and 3-methyl-pentanoic acid (Table 2). Part of the greater efficiency of the co-products as antimicrobials may be attributed to these increases. Furfural, for example, is a potent aldehyde derived from lignocellulosic biomass with a solid antimicrobial action [97,98,99]. In the wood-only WV, the furfural area percent was 8.98%, increasing to 23.12% in the co-products (Table 2). The antimicrobial action that furfural performs is linked to the toxicity mechanisms that this substance can exert on microbial cells. Furfural directly interferes with the energy metabolism of microbial cells. Additionally, it causes damage to cell DNA, inducing oxidative stress, interacting with the cell membrane of microorganisms, and causing changes in its permeability [100].

The cyclopentanones group increased in WVs during carbonization (Table 2). This table makes it possible to identify various species of these chemical groups. Their presence can result in an increasing antimicrobial action and they have the capacity to cause oxidative stress in microbial cells, reducing their viability [101,102]. The organic acids were another group of compounds that showed significant increases in their contents. For example, the percentage of acetic acid in the WV of eucalyptus wood was 2.45%, increasing to 6.19, 6.80, and 6.71 in the products from marjoram, Peruvian oregano, and thyme, respectively. This may have increased the antimicrobial action of these products. This acid’s antimicrobial activity as an antiseptic is well demonstrated in the literature [103,104,105,106,107,108].

A study shows that, in treatment against psychrotolerant strains, acetic acid resulted in inhibition at a concentration of 0.25%, emphasizing the high antimicrobial potential of this product [109]. Results demonstrate that this acid causes damage to the plasma membrane of microbial cells [110]. Another increase in organic acid is related to propanoic acid. This acid could inhibit the methicillin-resistant *S. aureus* strain’s growth [111], ruminal microorganisms during in vitro tests [112], *E. coli*, and *Salmonella* ssp. [113]. Furthermore, studies demonstrated that several propanoic acid derivatives play an essential inhibitory role against gram-positive, gram-negative, and fungal strains [114,115]. The same occurred with butanoic acid. This compound was absent in the WV produced only with eucalyptus wood, but its content can reach up to 2.3% in the co-products from herbs (Table 2). Butanoic acid efficiency has been demonstrated against several strains [116,117,118].

In general, the action mechanisms of organic acids against microbial strains are linked to the acidification of the intracellular environment and even of the environment external to the cells, resulting in damage to them. Another study [111] confirms this hypothesis, using propionic acid as a basis. Another form of action of organic acids is the changes caused to the microbial genetic material, which can be caused by acid stress [119,120]. Studies have shown that organic acids inhibited *Bacillus megaterium* and *Bacillus pumilus* [121,122]. Therefore, the better efficiency demonstrated by the co-products from herbs compared to eucalyptus WV can also be attributed to the greater concentration and variety of organic acids that resulted from the co-pyrolysis. Studies have shown that the association of different organic acids and higher concentrations can result in greater inhibitory efficiency against certain microbial strains [110,122]. Furthermore, in addition to the cases mentioned above, several other reactions of increased content of some chemical compounds can be observed in Table 2. Thus, it is suggested that the better action of the co-products is due, in part, to these increases, as they probably enhanced the original antimicrobial effect of WV.

In addition to the difference between the co-productions and the pure eucalyptus wood WV, there was a difference in action among co-products. As previously commented, this difference may be attributed to the chemical particularities of each product, since their chemical composition also varied significantly (Table 2). Among these co-products, the most efficient was the one from thyme. In contrast, Peruvian oregano was the co-production with the lowest efficiency (Table 2). There were at least 62 compounds, more or less, between the co-products compared to the eucalyptus WV. In addition, no single product gathered the highest percentages of the most promising compounds for antimicrobial action discussed in this research (Table 2). This becomes evident when comparing the difference in efficiency between the thyme and Peruvian oregano co-products (the most and the least efficient, respectively). The WV from thyme did not have the highest concentrations of the compounds. For example, the acetic acid content in the co-production with Peruvian oregano was higher than in thyme (Table 2). The same occurred with phenol, 2-methoxy-phenol, cyclopentanone, propanoic acid, etc. However, compared to the Peruvian oregano WV, the thyme product had higher contents of furfural, thymol, 2-cyclopenten-1-one, butanoic acid, crotonic acid, etc. (Table 2). These variations also occurred across the other products analyzed (rosemary, marjoram, and Turkish oregano). As such, the example using thyme and Peruvian oregano WVs was used to facilitate the discussion of the data. Thus, it is suggested that the greater efficiency of thyme WV cannot be attributed solely to the greater concentration of specific components but rather to the presence of these compounds in their composition and their joint action. Other studies suggest this same fact, indicating that the action of WV is attributed to the union of different compounds acting on microbial cells [21,27].

Even emphasizing the joint action of chemical compounds, it is worth highlighting that the WV from thyme presented a significantly higher furfural content than the other WVs, with 23.12%, against eucalyptus wood (8.92%), marjoram (10.52%), Peruvian oregano (11.32%), and Turkish oregano (19.98%), respectively, as shown in Table 2. This may have had a relevant impact on the antimicrobial action of the thyme co-product, given the effectiveness of furfural against microbial strains [97,98,99,100]. In addition to furfural, there is thymol. Thymol, the substance of interest in this research, emerged after co-productions with marjoram, Peruvian and Turkish oregano, and thyme. The highest content of this compound was found in thyme WV. Thus, it is assumed that this may have directly influenced the greater efficiency of action demonstrated by this WV. Thymol presents proven antimicrobial action [123,124,125]. Furthermore, the literature has demonstrated that this chemical is an adjuvant capable of enhancing the antimicrobial effect of some products. This is attributed to its ability to act synergistically with drugs, inhibiting the gene expression of resistance and virulence genes [45].

Another study demonstrates this synergy using the combination of thymol with oxacillin against resistant strains of *S. aureus*, reducing the microbial load, prolonging the effect of the antibiotic, and generating intracellular extravasation of the cells [63]. This combination also proves efficient against biofilm-forming strains. This is shown in one study [126], where the adjuvant action of thymol with ciprofloxacin destroyed *P. aeruginosa* biofilms. This justifies the assumption of the direct influence of thymol on the antimicrobial action that some WVs revealed. The integrated action of these different compounds against the same microorganism may hinder the emergence of microbial resistance, as [21,36] commented.

### 3.2. Results of Microbiological Assays

The experimental results show that all WVs exerted inhibitory efficiency against the assessed microorganisms (Table 3). Among the compounds found in the composition of the pyroligneous extract are phenolic compounds, organic acids, phenols, alcohols, ketones, aldehydes, and pyrans, among others [127,128]. Some of these compounds have their bioactive action established in the literature, and the antimicrobial action of some is already consolidated [95,129,130,131]. A clear example of the antimicrobial action of aldehydes present in WVs is the work of [111], where it was demonstrated that these constituents greatly influence WV’s antibacterial activity. The less expressive results were probably due to the absence of a compound such as carvacrol [132], which is common in aromatic herbs and is antibacterial. It should also highlight the capacity of the action of extracts associated with aromatic herbs for the fungal strain, where the presence of phenols [20] on the cell wall of the yeast of the genus *Candida* spp. [16], associated with guaiacol and furfural, represent approximately 60% of the composition of WV and are likely responsible for the antifungal action of WV [133].

In the present research, there was a difference in the efficiency of action among the WVs from co-pyrolysis against different microbial strains. This fact is expected and can occur due to a variety of factors, such as microbial resistance through modification of target sites, efflux pumps, and the formation of membrane vesicles that each specific strain can present [134,135,136], genetic variability between strains, acquired by vertical and horizontal transfer [137,138,139,140], and the chemical composition of the tested products, as their mechanisms of action may present different action against each microorganism [141,142,143]. Another factor that may have influenced this is the difference in the thickness and composition of the cell walls of the organisms evaluated [144]. Regarding bacterial strains, gram-positive and gram-negative strains may respond differently to the action of antimicrobials, and this is directly related to the particularities of the structure and composition of their cell walls [136,145,146,147]. The cell wall of gram-positive strains is generally composed of a thick layer of peptidoglycan and an internal plasma membrane [148]. In gram-negative cells, the conformation of the wall is different. They have two distinct protective membranes: an external one formed of lipopolysaccharides, a thinner, internal one, and a thin aqueous layer of peptidoglycan between them [149].

These different characteristics confer different reactions to the presence of antimicrobials. Gram-negative strains, due to the presence of this outer membrane, generally present more excellent resistance to antimicrobial drugs since, to access their target sites, the substances must first be able to penetrate this membrane that functions as a vital permeability barrier [150,151]. In addition, the defense mechanisms developed by gram-negative cells can also make them more resistant. An example of this is the limitation of influx, also associated with the outer membrane, since, to act on cells, many antimicrobials must penetrate this membrane. Thus, gram-negative strains have developed mechanisms to reduce the entry of these substances, reducing the influx by regulating the permeability of the outer membrane present in the cells [152], such as the bacterium *P. aeruginosa*. However, what was observed with the results of this research was that, in co-productions with herbs, the strains most sensitive to the action of the extracts were gram-negative strains, *A. baumannii* and *P. aeruginosa* (Table 3). These data demonstrate the antimicrobial potential that these co-productions present against gram-negative strains. This potential may be associated with the presence of a variety of different compounds acting on the same cell, which does not present sufficient forms of resistance to all compounds present in WVs; that is, even if the microbial cell can resist the action of a specific WV component, it is not able to protect itself against all of them [21,27].

Regarding the comparison between the co-produced WVs and the one from eucalyptus wood, it is worth noting that for the co-products, there was no MIC higher than 3.125%, unlike for the eucalyptus WV that had MICs of 6.25% against *E. coli*, *P. aeruginosa*, *P. aeruginosa* (PA01), and *S. aureus* (Table 3). This fact makes the co-products associated with herbs desirable since they can provide adjuvants that enhance antimicrobial activity. This difference in action found is probably associated with the chemical changes in the product when associated with herbs. This will be discussed in more detail in the next section. However, it can be anticipated that adding herbs during the pyrolysis process can lead to the formation of new chemical compounds since the final chemical composition of the product is directly linked to the material used for its production [27,153,154]. In addition, processing and association with other materials can also cause the increase, decrease, emergence, or disappearance of compounds already existing in the composition of the original product [81,84].

Among the co-products, the herb that demonstrated the best performance was thyme. Thyme WV presented the lowest MIC values (1.56%) for *A. baumanni*, *P. aeruginosa*, and *P. aeruginosa* PA01 (Table 3). This is probably associated with the final chemical composition of the WVs after association with the different herbs analyzed. This chemical composition presents thymol, which promotes the disintegration of gram-negative bacteria’s outer membrane and increases the cytoplasmic membrane’s permeability and depolarization [155], which will be discussed in more detail in the next topic. However, it can be anticipated that the chemical composition of the wood WV and the extracts or essential oils of the plants used in the research are different regarding specific chemical compounds [24,27,42,53,75,76]. A chemical component widely used as a bioactive molecule is thymol [74,156,157]. This component is found in thyme essential oil and appeared in the WV after the association with this plant in co-productions (Table 2). Among other substances, the better efficiency of action that thyme presented can also be attributed to the presence of thymol. Table 3 shows that when eucalyptus wood was combined with herbs, the components in both raw materials probably interacted, resulting in an even more chemically diverse product.

Regarding the trends expressed in Figure 2, it is observed that higher concentrations of WVs resulted in lower absorbance values, and as these concentrations decreased, the absorbance values increased. The absorbance or optical density (OD) of a microbial medium reflects the inhibitory potential of an antimicrobial agent since the higher the OD, the lower the antimicrobial efficiency in question. Therefore, more microbial cells could multiply in that medium, exposing the low inhibition capacity of the evaluated product [158,159]. Similar behaviors were demonstrated in a previous work [160], which tested the *Rhizophora apiculata* and [27] *Bambusa vulgaris* WVs. It was found that the greater the amount of WV used, the lower the OD of the medium.

## 4. Materials and Methods

### 4.1. Materials Collection

Wood from a *Eucalyptus urophylla* x *Eucalyptus grandis* hybrid (clone I144) was used to obtain the different types of WV. Harvesting logs from an 8-year plantation located in Macaíba, RN, Brazil (5° 51′ 36″ S, 35° 20′ 59″ W) provided the wood for the research. Wood test specimens were obtained using a standard method [24,65]. Wood disks 3.0 cm thick were collected and divided into four wedges each. The samples were dried in a forced air circulation oven (Sterilifer—SX cr/80, São Caetano do Sul, Brazil) at 103 + 2 °C for 48 h until reaching the bone-dry state. The aromatic herbs were *Origanum majorana* (marjoram), *Origanum vulgare* (Peruvian oregano), *Origanum onites* (Turkish oregano), *Rosmarinus officinalis* (rosemary), and *Thymus vulgaris* (thyme). All herbs in a granulometry of roughly 20 mesh were purchased in the Brazilian market (Atacadão Ervas—São Paulo, Brazil).

### 4.2. Carbonization Process and Pyrolysis Liquids Refining

Wood wedges were carbonized together with the aromatic herbs in a proportion of 25% (weight/weight) related to the bone-dry wood from ambient temperature until 450 °C, with a heating rate of 0.94 °C min^−1^. The ratio of wood/aromatic herbs was set in preliminary tests. The experiment was set according to an entirely randomized design, encompassing six experimental treatments, namely T1 (wood alone), T2 (wood + 25% of marjoram), T3 (wood + 25% of Peruvian oregano), T4 (wood + Turkish oregano), T5 (wood + 25% of rosemary), and T6 (wood + 25% of thyme). Treatment 1 was fitted out as the comparison reference. The different mixtures were placed inside a metallic cylinder and charred in a laboratory muffle furnace, following the procedures described by [24]. Five carbonization runs were conducted for each experimental treatment, and gravimetric yields in charcoal, pyrolysis liquids, and gases were determined for each run. The amounts of carbonized material ranged from 500 to 550 g per replicate. The smoke was driven to a water-cooled condenser at 25 °C during the carbonizations to yield the raw pyrolysis liquids (RPLs). The RPLs from each replicate of each treatment were combined to form a composite sample. They were subjected to vacuum distillation to achieve a refined WV for further chemical profiling and microbiological assays. Six different types of refined WV were obtained following the purification process described by [37]. The WVs were placed in amber flasks and stored under refrigeration (5 °C).

### 4.3. Chemical Profiling of WVs by Gas Chromatography and Mass Spectrometry

Firstly, 1.5 mL of concentrated ammonium hydroxide solution (Caledon, UN 2672, Canada) was added to 5 mL aliquots of the samples to increase the pH to around 7.0. Then, three 3 mL extractions were carried out using HPLV-grade ethyl acetate (Merck, São Paulo, Brazil). After the liquid-liquid extraction, 1 mL of the organic fraction with ethyl acetate was transferred to GC vials and promptly analyzed. The GC-MS analyses of the samples were carried out in a Shimadzu QP 2010 gas chromatograph/mass spectrometer (single quadrupole). The separation was performed in a DB-Wax 52 CB column (Agilent, São Paulo, Brazil) 30 m length, 0.25 mm diameter, and 0.25 μm film thickness), keeping the injector temperature at 250 °C. The samples (1 µL) were injected in a split ratio of 1:10, and the oven temperature program was as follows: 50 °C for 2 min with a heating rate of 2 °C min^−1^ from 50 to 240 °C, holding the final temperature for 2 min. Helium was used as carrier gas at a constant flow rate of 1 mL min−1. The total run time was 99 min. The MS acquisition range was from *m*/*z* 50 to 650 Da. The electron ionization source (EI) and interface of the MS were kept at 250 °C. The solvent cut time was 4 min. Major and minor compounds were detected and identified based on their characteristic electron ionization (EI, 70 eV) mass spectra by comparison with those in the NIST library. Most of the chemical compounds reported here had at least 85% mass spectrum similarity, but some minor compounds were reported with >80% similarity.

### 4.4. Microbiological Assays

The antimicrobial effect of the different WVs was assessed against strains of *Acinetobacter baumannii* (ATCC 19606), *Escherichia coli* (ATCC 23922), *Klebsiella pneumoniae* (ATCC 13883), *Pseudomonas aeruginosa* (ATCC 27853), *Pseudomonas aeruginosa* (PA01 1044), *Staphylococcus aureus* (P2340), *Staphylococcus aureus* (MRSA), and *Candida glabrata* (isolated from a cockatiel). All microorganisms were kept in BHI broth under refrigeration. All assays were conducted in triplicates, and the minimum inhibitory, bactericidal, and fungicidal concentrations were determined (respectively, MIC, MBC, and MFC). Analyses to determine the MIC were performed through the broth dilution method with 96-well microplates, following the procedures described in the M07-A9 protocol—Methodology for Dilution Antimicrobial Susceptibility Testing for Aerobically Growing Bacteria, from the Clinical and Laboratory Standards Institute [161]. All microbiological assays had chlorhexidine 0.2% (Vicfarma, São Paulo, SP, Brazil) as a reference.

Before each assay, the microorganisms were grown in BHI (brain heart infusion) broth at 37 °C ± 1 °C for 24 h in a laboratory bacteriological oven (FANEM, São Paulo, SP, Brazil). After incubation, inocula were pipetted and transferred to assay tubes containing the same sterile culture medium. Absorbances of the microorganism colonies were acquired using a UV–vis spectrophotometer (Biospectro SP-22 model, São Paulo, Brazil) before and after exposure to WVs to quantify their effect on the microbes’ growth at a wavelength of 530 nm. To confirm colonies’ densities, the reference value for the absorbance determination ranged from 0.08 to 0.1, corresponding to 0.5 (MacFarland number) [162].

To each microplate well, 100 µL of sterile BHI was added. Then, serial dilutions, always in triplicate, were performed by adding 100 µL of refined WV to the first well, totaling a final volume of 200 µL and resulting in the first dilution. After this, 100 µL of the initial solution was collected from the first well, and so on, so the concentrations started at 50 until 0.781% (respectively, 50, 25, 12.5, 6.25, 3.125, 1.563, and 0.781% of WV). Right after the inoculation, the absorbances were determined with an Elisa microplate reader (URIT Medical Electronic, Curitiba, PR, Brazil) at 580 nm for bacteria and 620 nm for *Candida glabrata*. Then, the microplates were incubated at 37 °C ± 1 °C for 24 h, and after this period, the absorbances were reread. After incubation, the microplates were carefully observed to visually determine the MIC, considering the first set of replicates presented themselves as utterly translucid to light. In other words, this translucid point was considered MIC.

Subsequently, the MBC and MFC tests were obtained. Aliquots of 50 μL were removed from the microwells where there was no visible microbial growth (MIC and WV concentrations higher than MIC) from each microplate well. The aliquots were superficially seeded on Petri dishes containing sterilized BHI. The plates were incubated following the same procedure used for the MIC. After incubation, the presence or absence of microbial colonies was verified. The MBC and MFC were defined as the lowest WV concentrations capable of causing total inactivation of the microorganisms. The total lack of growth of microbial colonies in the agar determined this inactivation. The absorbance data obtained after the spectrophotometer readings were processed in a Microsoft Excel 2016 [163] spreadsheet to display the data in graphs.

## 5. Conclusions

It was concluded that all WVs analyzed presented efficient antimicrobial potential. The co-productions enhanced the antimicrobial action of the WV of *E. urograndis*. The most susceptible microbial strains were *A. baumanni* and *P. aeruginosa* in products co-produced with 25% herbs. The most efficient co-production was the one carried out in association with thyme. On the other hand, the co-production with Peruvian oregano demonstrated the least efficiency compared to the tested strains. However, this does not mean this product does not work, since the MICs obtained were satisfactory values of 3.125%. Thymol was incorporated into the WV after co-productions with marjoram, Peruvian and Turkish oregano, and thyme. The co-product with the highest thymol content was the WV from thyme. After the co-production process, there were significant changes in the chemical constitution of the products. However, it is observed that large groups of the WV base compounds remained, these being phenolic compounds, aldehydes, furans, organic acids, etc.

Therefore, the greater efficiency of action demonstrated by the co-productions is attributed to the increase and incorporation of some chemical compounds in the chemical composition of the WVs. Furthermore, the more significant activity shown by the co-production with thyme is attributed to the variety of chemical components of the product and the increased content of some of these components, in addition to the adjuvant action that thymol may have played. Thus, it is suggested that the antimicrobial action demonstrated by the WVs cannot be attributed to the occurrence of an isolated compound but rather to the set of compounds acting simultaneously on the microorganisms. Further research is necessary to understand each WV component’s role in this product’s bioactivity, aiming at more excellent knowledge of this role in their joint action. Finally, it is concluded that the co-production of WVs with aromatic herbs is a viable technological alternative for extracting relevant chemical compounds from these herbs, increasing extraction efficiency and reducing its cost.

## Figures and Tables

**Table 1:** Gravimetric yields of the carbonization runs.

	Gravimetric Yields (%)		
Material	Charcoal	Liquids	Gases
Eucalyptus wood	34.9	43.1	22.0
Wood + marjoram	33.8	41.1	25.1
Wood + Peruvian oregano	33.4	42.1	24.5
Wood + Turkish oregano	33.3	42.7	24.0
Wood + rosemary	32.8	40.5	26.7
Wood + thyme	34.5	41.7	23.8

Gravimetric yields in charcoal, pyrolysis liquids, and gases calculated based on the initial weight of bone-dry wood and bone-dry wood + herbs.

**Table 2:** GC/MS annotated compounds in eucalyptus wood WV and eucalyptus wood + aromatic.

	Eucalyptus Wood	Marjoram	Peruvian Oregano	Turkish Oregano	Rosemary	Thyme
Numbered Compounds	Relative Peak Areas (%)					
**(1) Furfural**	**8.92**	**10.52**	**11.32**	**19.98**	**19.61**	**23.12**
**(2) 2-methoxy-phenol**	**12.35**	**11.95**	**10.83**	**11**	**12.77**	**11.03**
**(3) Phenol**	**9.34**	*	**7.46**	**5.46**	**5.22**	*
**(4) Borazine, 2,4-dimethyl-**	*	**4.95**	*	*	*	**3.8**
**(5) 2-Furancarboxaldehyde, 5-methyl-**	**3.49**	**3.46**	**3.88**	**4.32**	**4.92**	**3.78**
**(6) Phenol, 2-methoxy-4-methyl-**	**5.98**	**4.07**	**3.89**	**4.33**	**4.03**	**3.08**
**(7) 2-Amino-1,3-propanediol**	*	*	*	**3.91**	**4.97**	*
**(8) 2-Cyclopenten-1-one**	**1.87**	**3.55**	**2.81**	**3.98**	**3.22**	**3.91**
**(9) 2-Cyclopenten-1-one, 2-methyl-**	0.95	**3.29**	**2.80**	**3.43**	**2.43**	**3.03**
**(10) Hexanoic acid, 6-bromo-**	**2.47**	*	*	*	*	*
**(11) Acetic acid**	**2.45**	**6.19**	**6.80**	*	*	**6.71**
**(12) 1-Hydroxy-2-butanone**	0.82	**2.22**	1.42	**2.5**	**1.82**	**2.24**
**(13) Butanoic acid**	*	**2.11**	1.34	1.17	**2.3**	**1.92**
**(14) 2-Cyclopenten-1-one, 3-methyl-**	**2.36**	**2.28**	**1.88**	1.21	1.41	**1.6**
**(15) 2-Cyclopenten-1-one, 16) (16) 2,3-dimethyl-**	**1.88**	**1.83**	**1.60**	**1.67**	**1.96**	1.47
**Cyclopentanone**	*	**1.79**	**1.90**	**1.59**	**2.1**	**1.81**
**(17) Phenol, 4-ethyl-2-methoxy-**	*	*	*	*	**1.73**	*
**(18) Methyl 2-furoate**	*	**1.55**	1.38	1.37	1.41	1.24
**(19) Propanoic acid**	1.43	**1.85**	**2.41**	**1.61**	**1.99**	**2.29**
**(20) Phenol, 3-methyl-**	**3.87**	1.17	1.26	0.93	1.04	0.92
(21) Bicyclo[2.2.1]heptan-2-one, 1,7,7-trimethyl-, (1R)-	*	*	*	*	1.11	*
(22) 2-Cyclopenten-1-one, 2,3-dimethyl-	*	1.24	1.03	1.21	0.85	1.07
**(23) Phenol, 4-methyl-**	**2.58**	0.97	**2.57**	1.36	0.97	0.56
(24) 2-Butanone, 1-(acetyloxy)-	*	0.92	0.94	*	*	*
(25) 3-Hexen-2-one, 3-methyl-	*	*	0.91	0.81	*	*
(26) Phenol, 3,4-dimethyl-	0.8	*	*	*	*	*
**(27) 1,2-Cyclopentanedione, 3-methyl-**	**2.18**	0.78	0.64	0.51	0.38	0.46
(28) N-Nitrosodimethylamine	0.09	0.8	0.41	0.78	0.48	0.6
(29) Phenol, 2-methoxy-3-methyl-	0.16	0.77	0.71	0.61	0.45	0.53
(30) 2-Cyclopenten-1-one, 3,4-dimethyl-	0.44	0.72	0.77	1.02	*	0.77
(31) 2,3-Pentanedione	0.57	0.73	0.98	0.15	0.07	0.32
(32) 3-(diethylamino)-1,2-propanediol	0.11	0.71	0.46	*	*	*
(33) Pyridine	1.02	0.69	*	0.34	0.13	*
**(34) Phenol, 2,6-dimethoxy-**	**10.3**	0.7	0.6	0.2	*	0.2
(35) 2-Furanmethanol	*	0.64	0.50	*	*	*
(36) 2-Cyclopenten-1-one, 3-ethyl-	0.55	0.56	0.49	0.4	0.36	0.44
(37) 2,6-Heptadien-1-ol, 2,4-dimethyl-	0.56	*	*	*	*	*
(38) 3-Cyclohexen-1-ol, 4-methyl-1-(1-methylethyl)-, (R)-	*	0.54	0.87	0.16	*	*
(39) 1-Buten-3-yne, 1-(1,1-dimethylethoxy)-, (E)-	*	*	*	*	*	0.54
(40) 2-Butanone, 1-(acetyloxy)-	0.51	*	*	*	*	*
(41) 2-Cyclopenten-1-one, 2,3,4-trimethyl-	0.10	0.51	0.44	0.51	0.5	0.47
(42) 2-Butenal, 2-ethyl-	0.48	*	*	*	*	*
(43) Bicyclo[2.2.1]heptan-2-ol, 1,7,7-trimethyl-, (1S-endo)-	*	*	*	*	0.48	*
(44) 2-Methoxy-5-methylphenol	*	0.47	0.48	*	0.27	0.38
(45) Furan, 2-(methoxymethyl)-	*	0.46	0.40	0.08	*	0.25
(46) Phenol, 2,4-dimethyl-	*	0.58	*	0.45	0.48	0.51
(47) 1,2,3-Trimethoxybenzene	0.45	*	*	*	0.15	*
(48) Bicyclo[2.2.2]octane, 2-methyl-	0.17	0.43	*	*	0.37	0.36
(49) 4,4-Dimethyl-2-cyclopenten-1-one	*	*	*	*	0.42	*
(50) Ethanone, 1-(1-cyclohexen-1-yl)-	*	*	*	*	0.41	*
(51) Phenol, 2,6-dimethyl-	0.08	0.4	0.38	0.32	0.28	0.32
(52) 3-Ethenyl-3-methyl cyclopentanone	*	0.4	0.27	0.22	*	0.38
(53) 5-Octyn-4-one, 2,2,7,7-tetramethyl-	*	*	*	0.4	*	*
(54) Butanoic acid, 2-hydroxy-, methyl ester	*	0.4	0.23	*	*	0.18
(55) Pentanoic acid, 3-methyl-	0.39	0.84	0.86	0.66	0.48	0.59
(56) 2-Cyclopenten-1-one, 2,3-dimethyl-	*	0.39	0.34	0.33	0.22	0.35
(57) 2-Cyclopenten-1-one, 3-ethyl-2-hydroxy-	0.20	0.38	0.33	0.29	0.11	0.33
(58) Cyclohexene, 1-isopentyl-	*	0.38	*	*	*	*
(59) (R)-(+)-3-Methylcyclopentanone	*	0.38	0.23	*	0.35	*
(60) Phenol, 2-methoxy-4-methyl-	0.40	*	*	0.36	*	*
(61) 2-Cyclopenten-1-one, 3-ethyl-2-hydroxy-	0.73	0.36	0.33	0.16	0.17	0.18
(62) 5,9-Dodecadien-2-one, 6,10-dimethyl-, (E,E))-	*	*	*	*	0.36	*
(63) Pentanoic acid, 4-methyl-	*	0.35	0.32	0.25	0.15	0.17
(64) 2,3-Dimethoxytoluene	*	0.35	0.27	0.28	*	0.28
(65) Bicyclo[3.3.1]nonane	*	*	0.35	0.43	*	*
(66) 1,3-Hexadiene, 3-ethyl-2-methyl-, (Z)-	*	0.34	0.28	0.25	*	*
(67) Cyclopentanone, 2-methyl-	*	0.33	0.26	0.39	0.34	0.32
(68) Ethylbenzene	0.43	0.33	*	0.27	0.35	0.23
(69) Phenol, 3,5-dimethyl-	0.34	0.47	0.44	0.31	0.22	0.42
(70) Cycloheptanone, 2-ethyl-	0.31	*	*	*	*	0.05
(71) Pentanoic acid	0.38	0.31	0.35	0.32	0.32	0.28
(72) 2,4-Pentadien-1-ol, 3-propyl-, (2Z)-	0.30	*	*	*	*	*
(73) Pyrazine, methyl-	*	0.29	*	*	*	*
(74) Adrenalone	*	0.28	0.30	0.25	*	0.2
(75) 2-Acetyl-5-methylfuran	0.29	0.28	0.30	0.33	0.35	0.22
(76) 3-Cyclohexene-1-carboxaldehyde, 1-methyl-	*	*	*	*	*	0.28
(77) 2,5-Hexanedione	0.28	*	*	*	*	*
(78) Vinyl butyrate	*	0.28	0.21	0.26	0.32	0.28
(79) 2-Butanol, 1-methoxy-	0.07	*	0.28	*	*	*
(80) Cyclohexaneacetic acid, α-ethyl-	*	0.28	0.25	0.37	*	0.26
(81) Phenol, 2-methoxy-4-propyl-	0.21	0.27	0.27	0.24	0.15	0.22
(82) 1,4-Dioxin, 2,3-dihydro-	0.05	0.26	0.19	0.25	0.28	0.24
(83) Cyclopentanone, 3-methyl-	*	*	*	0.26	*	0.26
(84) 2-Hydroxy-3-pentanone	*	*	0.25	*	0.32	0.24
(85) Phenol, 2-methyl-5-(1-methylethyl)-	*	*	0.2	0.2	*	0.1
(86) 2,3-Dimethoxytoluene	*	*	*	*	0.24	*
(87) 2-Hepten-3-ol, 4,5-dimethyl-	0.24	*	*	*	*	*
(88) 2-Cyclohexen-1-one	0.07	0.24	0.24	0.28	0.25	0.22
(89) Oxalic acid, isobutyl pentyl ester	*	0.24	*	0.4	*	*
(90) 3-Pentanol	*	0.24	*	0.46	0.41	0.27
(91) 2-Cyclopenten-1-one, 3-methyl-	0.04	*	0.24	0.27	*	0.28
(92) Cyclohexanone, 3-ethenyl-	*	*	*	*	0.23	*
(93) Pyrazole-4-carboxaldehyde, 1-methyl-	*	*	*	*	0.36	0.23
(94) 3-Furaldehyde	*	0.23	0.23	*	*	0.34
(95) 2-Propen-1-ol	*	0.25	0.23	0.23	0.29	0.2
(96) 4-Hepten-3-one, 4-methyl-	*	0.22	*	0.16	*	0.19
(97) Maltol	0.21	*	*	*	*	*
(98) Furan, 2,5-dihydro-3,4-dimethyl-	*	0.21	0.22	0.21	0.27	0.15
(99) 2(3H)-Furanone, dihydro-5-methyl-	0.21	*	*	*	*	*
(100) 1H-Imidazole-1-carboxamide, N,N-diethyl-	*	*	0.21	*	*	0.11
(101) Pyridine, 2-methyl-	0.07	0.21	*	0.11	*	*
**(102) Eucalyptol**	*	0.21	0.19	0.3	**1.63**	*
(103) Phenol, 2-ethyl-	0.09	0.2	0.29	0.19	0.16	0.17
(104) 2-Cyclopenten-1-one,3-ethyl-2-hydroxy-	0.57	0.2	0.20	0.2	0.19	0.21
(105) 3(2H)-Furanone, dihydro-2-methyl-	*	*	*	0.2	0.15	*
(106) 2,3-Pentanedione	0.03	0.2	0.24	0.19	0.11	0.32
(107) 1,3-Cyclopentanedione, 2-ethyl-2-methyl-	*	*	*	*	0.19	*
(108) 2-Hydroxy-3-propyl-2-cyclopenten-1-one	0.19	*	*	*	0.04	*
(109) 4-Hexen-3-one, 4,5-dimethyl-	0.31	*	0.19	*	0.22	*
(110) Butyrolactone	0.39	0.19	0.12	0.11	0.15	0.12
(111) 2-Cyclohexen-1-one, 2-methyl-	*	0.19	0.17	0.15	0.11	0.15
(112) 3-Heptyne	*	0.19	0.14	0.15	*	0.16
(113) Benzene, 1,3-dimethyl-	0.19	*	*	*	*	*
(114) Cyclooctene	*	0.18	0.16	*	*	0.13
(115) Uracil, 1-methyl-	*	*	*	0.18	0.13	*
(116) 2-Hexanol, 2-methyl-	*	0.18	0.16	0.18	0.22	0.18
(117) 3-Penten-2-one, (E)-	*	*	0.18	*	*	*
(118) 1,2,3-Trimethoxybenzene	0.2	*	*	*	*	*
(119) Cyclohexane, (1-methylethylidene)-	0.17	*	*	*	*	*
(120) Thymol	*	0.2	0.3	0.1	*	0.7
**(121) Ethanone, 1-(2-furanyl)-**	1.08	**3.72**	**3.53**	**3.62**	**3.68**	**2.94**
(122) 2H-Pyran-3(4H)-one, dihydro-	0.08	0.16	0.10	0.14	0.14	*
(123) p-Xylene	*	0.16	*	0.13	0.15	*
(124) Pentanoic acid, 3-methyl-	*	0.15	0.15	0.12	0.07	0.09
(125) Crotonic acid	*	*	0.19	0.15	0.13	0.22
(126) 2-Cyclopenten-1-one, 3-ethyl-2-hydroxy-	0.15	*	*	*	*	*
(127) 3-Penten-2-ol	0.15	*	*	*	*	*
(128) Cyclohexylmethyl formate	*	*	*	*	0.15	*
(129) 1,4-Pentanediamine	*	*	*	*	0.15	*

The table asterisks (*) mean the compound was not annotated in the respective WV. Major compounds are in bold to highlight them (area % higher than 1.5%).

**Table 3:** MIC, MBC, and MFC values were determined for the strains against which the WVs had their antimicrobial effect assessed.

Microorganism	Parameter	Eucalyptus WV	Marjoram	Peruvian Oregano	Turkish Oregano	Rosemary	Thyme
*Acinetobacter baumanni*	MIC	3.125	1.56	3.125	1.56	1.56	1.56
	MBC	6.25	3.125	3.125	3.125	3.125	3.125
*Escherichia coli*	MIC	6.25	3.125	3.125	3.125	3.125	3.125
	MBC	6.25	6.25	6.25	12.5	3.125	6.25
*Klebsiella pneumoniae*	MIC	3.125	3.125	3.125	3.125	3.125	3.125
	MBC	6.25	12.5	6.25	6.25	12.5	12.5
*Pseudomonas aeruginosa*	MIC	6.25	1.56	3.125	1.56	1.56	1.56
	MBC	6.25	3.125	3.125	3.125	6.25	3.125
*P. aeruginosa (PA01)*	MIC	6.25	3.125	3.125	3.125	3.125	1.56
	MBC	6.25	12.5	12.5	12.5	3.125	3.125
*Staphylococcus aureus*	MIC	6.25	3.125	3.125	3.125	3.125	3.125
	MBC	12.5	12.5	6.25	12.5	6.25	6.25
*S. aureus (MRSA)*	MIC	3.125	3.125	3.125	3.125	3.125	3.125
	MBC	6.25	12.5	12.5	3.125	6.25	3.125
*Candida glabrata*	MIC	3.125	3.125	3.125	3.125	3.125	3.125
	MFC	6.25	12.5	6.25	12.5	3.125	6.25

MIC = minimum inhibitory concentration (%); MBC = minimum bactericide concentration (%); MFC = minimum fungicide concentration (%).

## Data Availability

The raw data supporting the conclusions of this article will be made available by the authors on request.
